# Determination of the Accuracy of Transcerebellar Diameter in Estimating Gestational Age in the Second and Third Trimesters of Pregnancy

**DOI:** 10.7759/cureus.63292

**Published:** 2024-06-27

**Authors:** Satyanarayana Kummari, Vidhya Selvam, Preethi B

**Affiliations:** 1 Department of Radiology, All India Institute of Medical Sciences, Nagpur, Nagpur, IND; 2 Department of Obstetrics and Gynecology, Sree Balaji Medical College and Hospital, Chennai, IND

**Keywords:** ultrasonography (usg), second and third trimester, femur length, abdominal circumference, head circumference, biparietal diameter, foetal growth, small for gestational age (sga), appropriate for gestational age (aga), transcerebellar diameter

## Abstract

Background

Every antenatal woman and her treating doctor aim for a healthy newborn. In obstetrics, accurately determining the gestational age (GA) is a critical aspect of managing pregnancy and evaluating fetal growth and development. The transcerebellar diameter (TCD) is the greatest transverse measurement of the fetal cerebellum. The growth of the cerebellum is minimally affected by fluctuations in growth, making the TCD the most reliable measurement for predicting GA. The purpose of the present research is to determine the accuracy of TCD in estimating GA in the second and third trimesters of pregnancy.

Materials and methods

The study included 500 antenatal women at 18-40 weeks of gestation. We also measured TCD in addition to routine ultrasound parameters like biparietal diameter (BPD), head circumference (HC), abdominal circumference (AC), and femur length (FL). We used IBM SPSS Statistics for Windows, Version 22 (Released 2013; IBM Corp., Armonk, New York, United States) for statistical analysis. The collected data was subjected to statistical tests, including Pearson's correlation coefficient and coefficient of determination. We conducted a regression analysis and used correlation coefficients to compare each ultrasound-measured parameter with the GA.

Results

The current research demonstrates a significant linear relationship between the TCD and GA (r = 0.9865; p = 0.0001), a strong association between BPD and GA (r = 0.9541; p = 0.0001), between HC and GA (r = 0.9613; p = 0.0001), between AC and GA (r = 0.9489; p = 0.0001), and between FL and GA (r = 0.9697; p = 0.0001). TCD showed the best correlation with GA among all the biometric parameters. TCD showed a correct assessment of GA by the last menstrual period (LMP) in 479 (95.8%) antenatal women.

Conclusion

The current research concludes that the TCD can be utilized as an independent measure to determine GA in the second and third trimesters of pregnancy, particularly in cases where the LMP is unknown, no dating scan has been performed in the first trimester, initial assessment taking place in the third trimester and in fetuses with variations in head shape such as dolichocephaly and brachycephaly.

## Introduction

Every antenatal woman and her treating doctor aim for a healthy newborn. In obstetrics, accurately determining the gestational age (GA) is a critical aspect of managing pregnancy and evaluating fetal growth and development. Precision in this estimation is vital, as errors can lead to either preterm or post-term delivery. Moreover, in cases where the estimated date of delivery (EDD) is uncertain, there is a considerably higher likelihood of perinatal mortality [1-3]. Initially, clinicians used a combination of oral information about the last menstrual period (LMP) and physical examination to assess GA. Many antenatal women often present with an unclear LMP, without GA confirmation in the first trimester, and initial assessment taking place in the third trimester. Uncertainty regarding the GA could complicate the differentiation between fetuses with normal growth and those with growth abnormalities [3-6].

Given the unreliability of clinical assessment of fetal growth and the unclear LMP, prenatal ultrasonography offers a more precise method for assessing fetal growth. Ultrasonography plays a vital role in differentiating fetuses with normal growth from those with growth restriction. Ultrasound (USG) assessment of fetal biometry has emerged as a crucial and easily accessible technique for ensuring the normal growth of the fetus and accurately determining the GA [4].

Biparietal diameter (BPD), head circumference (HC), abdominal circumference (AC), and femur length (FL) are the measurements that are utilized more frequently in the process of assessing the growth of the fetus. However, these factors can only be correlated accurately when the GA is exactly determined. The challenge of distinguishing between fetuses that are appropriately sized for their GA (AGA) and those that are smaller for their GA (SGA) becomes difficult when there is uncertainty regarding the GA of the fetus [2,4].

The transcerebellar diameter (TCD) is the greatest transverse measurement of the fetal cerebellum. The fetal cerebellum can be observed through USG as early as 10-11 weeks of pregnancy. It exhibits a linear correlation with GA from the second trimester onward, meaning it grows in proportion to the stage of pregnancy. The posterior cranial fossa, which houses the fetal cerebellar hemispheres, is impervious to external forces [7,8]. Decreased uteroplacental blood flow is the main cause of intrauterine growth restriction (IUGR). In acute asphyxia, blood circulation to the cerebellum is maintained due to the redistribution of cardiac output to central regions of the body, such as the brain, heart, and adrenal glands. It has been suggested that the TCD is minimally affected by fetal growth restriction because of the brain-sparing effect that occurs throughout gestation. The growth of the cerebellum is minimally affected by fluctuations in growth, making the TCD the most reliable measurement for predicting GA. Measurement of TCD is a precise technique for assessing the GA in situations where the LMP is unknown, no dating scan has been performed, there are variations in the shape of the fetal head such as brachycephaly and dolichocephaly, and in fetuses with IUGR [8-10].

To our knowledge, there are only a few studies with 500 antenatal women determining the accuracy of TCD in estimating GA in pregnancy in south India. The purpose of the current research is to determine the accuracy of TCD in estimating GA in the second and third trimesters of pregnancy.

## Materials and methods

Five hundred antenatal women at 18-40 weeks of gestation, referred to the department of radiology at Great Eastern Medical School and Hospital (GEMS) located in Srikakulam, India, for USG scans were included in the research. The research was a prospective observational, cross-sectional study. The research was conducted for one year. Written informed assent was obtained from all pregnant women prior to the examinations. The research obtained approval from the Institutional Human Ethics Committee (IHEC) of Great Eastern Medical School and Hospital (GEMS).

Inclusion criteria

Antenatal women with excellent dates, at 18-40 weeks of gestation, and carrying a live singleton pregnancy were added to the research.

Exclusion criteria

Antenatal women with anomalous babies, multiple gestations, unreliable dates, and those suspected to have IUGR were excluded from the research.

Study procedure

**Figure 1:**
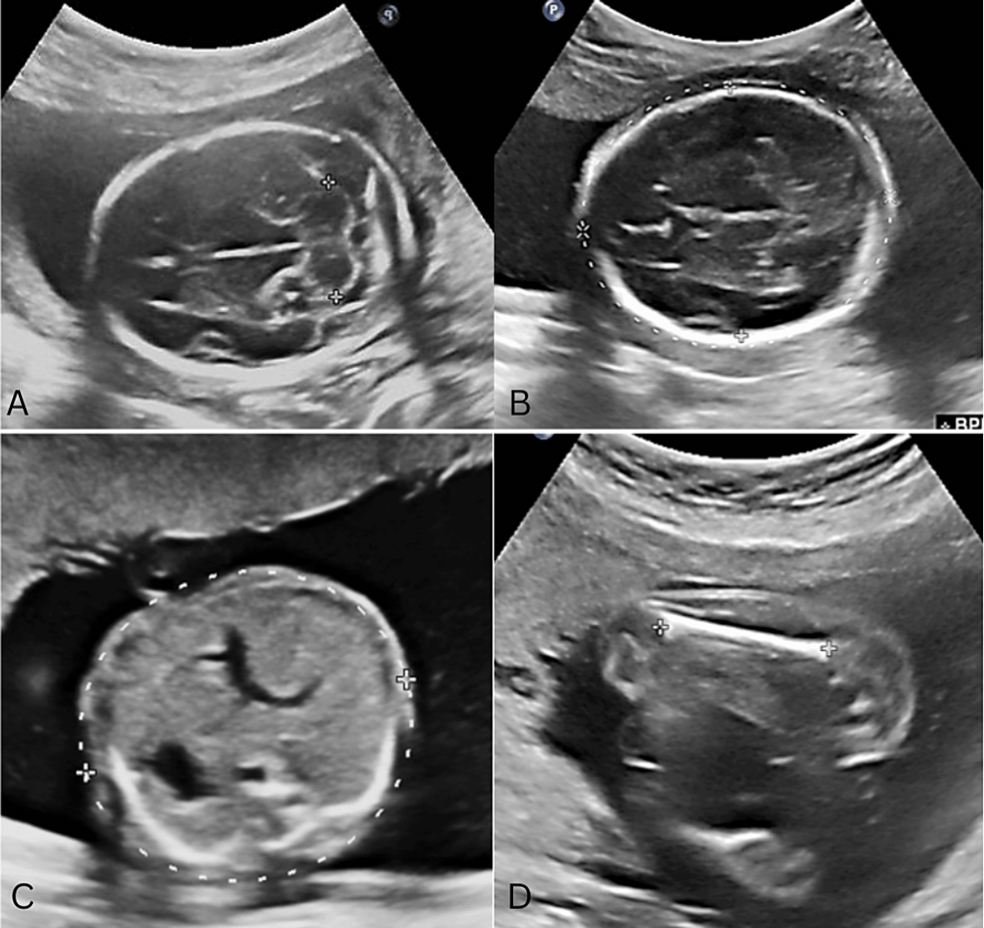
Fetal biometry. Ultrasonographic measurement of (A) TCD, (B) BPD and HC, (C) AC, and (D) FL TCD: transcerebellar diameter; BPD: biparietal diameter; HC: head circumference; AC: abdominal circumference; FL: femur length

Data collection employed a questionnaire and USG scan reports. The measurements were taken by a single radiologist to reduce inter-observer variability. Two measurements were averaged to reduce intra-observer variability. We also measured TCD in addition to routine USG parameters like BPD, HC, AC, and FL. Following the completion of the aforementioned measurements, the GA of the fetus was determined by utilizing the USG machine in accordance with the Hadlock table. All of the parameters that were measured, including BPD, AC, HC, FL, and TCD, were measured in millimeters, and the duration of pregnancy was recorded in weeks, with the nearest menstrual week being used as the reference point (Figure 1).

Statistical analysis

The collected data was imported into Microsoft Excel 2019 and subjected to statistical tests, including Pearson's correlation coefficient and coefficient of determination. We used IBM SPSS Statistics for Windows, Version 22 (Released 2013; IBM Corp., Armonk, New York, United States) to carry out the statistical analysis. We performed a regression analysis to determine the relationship between various parameters measured using ultrasonography, including BPD, HC, AC, FL, and TCD, and the GA of the fetus. We also employed correlation coefficients to determine the relationship between each ultrasound-measured parameter and the GA. Categorical data were encoded with numerical values and percentages, while quantitative variables were represented as the mean ± standard deviation (SD). We established a significance level of p = 0.05 to determine the statistical significance of a value. Any value less than or equal to 0.05 was deemed statistically significant.

## Results

Five hundred antenatal women at 18-40 weeks of gestation were added to the study. The women's ages ranged from 20 to 40 years, with an average age of 26.52 ± 4.31 years (Table 1).

**Table 1:** Age distribution in antenatal women

Age (in years)	Frequency	Percentage
20-25	202	40.4
26-30	242	48.4
31-35	45	9.0
36-40	11	2.2
Total	500	100

Out of 500 antenatal women, 352 (70.4%) were primigravidae and 148 (29.6%) were multigravidae. The mean birth weight and GA of the babies at delivery were 2.98 kg and 37.6 weeks, respectively (Table 2).

**Table 2:** Maternal variable distribution

Parity of the antenatal women	Frequency	Percentage
Primigravida	352	70.4
Multigravida	148	29.6
Total	500	100

Out of 500 antenatal women, 157 (31.4%) were between 18 and 22 weeks of gestation, followed by 149 (29.8%) who were between 28 and 32 weeks of gestation. There is a linear relationship between TCD and advancing GA in fetuses with normal growth. The distribution of the mean transcerebellar diameter in the population is given in Table 3.

**Table 3:** Distribution of the mean transcerebellar diameter in the population GA: gestational age; TCD: transcerebellar diameter; SD: standard deviation

GA (in weeks)	TCD (mm) mean ± SD	Frequency	Percentage
18	18.5 ± 0.4	25	5.0
19	20.7 ± 0.3	35	7.0
20	21.1 ± 0.2	40	8.0
21	21.8 ± 0.2	25	5.0
22	22.6 ± 0.3	32	6.4
23	24.3 ± 0.3	15	3.0
24	25.2 ± 0.4	10	2.0
25	27.0 ± 0.3	8	1.6
26	29.5 ± 0.4	10	2.0
27	30.3 ± 0.4	11	2.2
28	33.4 ± 0.5	48	9.6
29	34.8 ± 1.2	24	4.8
30	35.6 ± 1.5	22	4.4
31	37.2 ± 1.5	14	2.8
32	38.4 ± 1.6	41	8.2
33	40.2 ± 1.5	22	4.4
34	42.4 ± 1.5	10	2.0
35	43.8 ± 1.6	14	2.8
36	45.6 ± 1.8	35	7.0
37	47.4 ± 1.8	11	2.2
38	48.5 ± 1.9	30	6.0
39	50.2 ± 2.2	10	2.0
40	51.5 ± 2.5	8	1.6
Total		500	100

The study shows a substantial linear relationship between the TCD and GA (r = 0.9865; p = 0.0001), a strong association between BPD and GA (r = 0.9541; p = 0.0001), between HC and GA (r = 0.9613; p = 0.0001), between AC and GA (r = 0.9489; p = 0.0001), and between FL and GA (r = 0.9697; p = 0.0001). Among all the biometric parameters, TCD was seen to demonstrate the best correlation with GA (Table 4).

**Table 4:** Correlation between GA from LMP and different fetal variables GA: gestational age; LMP: last menstrual period; BPD: biparietal diameter; HC: head circumference; AC: abdominal circumference; FL: femur length; TCD: transcerebellar diameter

GA from LMP	Parameters	Correlation coefficient (r) value	p-value
	Correlation with BPD	0.9541	0.0001
	Correlation with HC	0.9613	0.0001
	Correlation with AC	0.9489	0.0001
	Correlation with FL	0.9697	0.0001
	Correlation with TCD	0.9865	0.0001

The study shows a substantial linear association between the TCD and GA (r^2 ^= 0.9731; p = 0.0001), a strong association between BPD and GA (r^2 ^= 0.9103; p = 0.0001), between HC and GA (r^2 ^= 0.9240; p = 0.0001), between AC and GA (r^2 ^= 0.9004; p = 0.0001), and between FL and GA (r^2 ^= 0.9403; p = 0.0001). Among all the biometric parameters, TCD was seen to demonstrate the best correlation with GA (Table 5).

**Table 5:** Correlation between GA from LMP and different fetal variables GA: gestational age; LMP: last menstrual period; BPD: biparietal diameter; HC: head circumference; AC: abdominal circumference; FL: femur length; TCD: transcerebellar diameter

GA from LMP	Parameters	Coefficient of determination (r^2^) value	p-value
	Correlation with BPD	0.9103	0.0001
	Correlation with HC	0.9240	0.0001
	Correlation with AC	0.9004	0.0001
	Correlation with FL	0.9403	0.0001
	Correlation with TCD	0.9731	0.0001

The study shows a strong association between TCD and BPD (r = 0.9731; p = 0.0001), between TCD and HC (r = 0.9643; p = 0.0001), between TCD and AC (r = 0.9617; p = 0.0001), and between TCD and FL (r = 0.9868; p = 0.0001). Among these parameters, FL shows a good correlation (r = 0.9868; p = 0.0001) with TCD (Table 6).

**Table 6:** Correlation between TCD and different fetal variables TCD: transcerebellar diameter; BPD: biparietal diameter; HC: head circumference; AC: abdominal circumference; FL: femur length

TCD	Parameters	Correlation coefficient (r)-value	p-value
	Correlation with BPD	0.9731	0.0001
	Correlation with HC	0.9643	0.0001
	Correlation with AC	0.9617	0.0001
	Correlation with FL	0.9868	0.0001

In the current research, out of 500 antenatal women, TCD showed a correct assessment of GA by LMP in 479 (95.8%) antenatal women.

## Discussion

In obstetrics, accurately determining the GA is a critical aspect of managing pregnancy and evaluating fetal growth and development. Furthermore, when the expected date of delivery is unclear, it can result in either preterm or post-term delivery, and there is a considerably higher likelihood of perinatal mortality [1-3]. BPD, HC, AC, and FL are the parameters that are utilized more frequently in the process of assessing the growth of the fetus (Figure 1). However, these factors can only be correlated accurately when the GA is exactly determined. Furthermore, to accurately determine the GA, it is crucial to have precise information about the LMP or undergo a dating scan in the first trimester. Many antenatal women often present with an unclear LMP, without GA confirmation in the first trimester, and initial assessment taking place in the third trimester [1,3]. The TCD is the greatest transverse measurement of the fetal cerebellum. TCD measurements are reliable for determining true GA in situations where the LMP is not known, and a dating scan was not performed during the first trimester [11-14].

In the current research, most of the antenatal women, 242 (48.4%) out of 500, were varied between 26 and 30 years, with a mean age of 26.52 ± 4.31 years (Table 1). Out of 500 antenatal women, 352 (70.4%) were primigravidae and 148 (29.6%) were multigravida. The mean birth weight and GA of the babies at delivery were 2.98 kg and 37.6 weeks, respectively (Table 2). Most of the antenatal women, 157 (31.4%), were between 18 and 22 weeks of gestation, followed by 149 (29.8%) who were between 28 and 32 weeks of gestation. There is a linear relationship between TCD and advancing GA in fetuses with normal growth. The distribution of mean TCD in the population is given in Table 3.

The current research demonstrates a significant linear relationship between the TCD and GA (r = 0.9865; p = 0.0001), a strong association between BPD and GA (r = 0.9541; p = 0.0001), between HC and GA (r = 0.9613; p = 0.0001), between AC and GA (r = 0.9489; p = 0.0001), and between FL and GA (r = 0.9697; p = 0.0001) (Table 4). There was significant linear association between the TCD and GA (r^2 ^= 0.9731; p = 0.0001), a strong association between BPD and GA (r^2 ^= 0.9103; p = 0.0001), between HC and GA (r^2 ^= 0.9240; p = 0.0001), between AC and GA (r^2 ^= 0.9004; p = 0.0001), and between FL and GA (r^2 ^= 0.9403; p = 0.0001) (Table 5). Among all the biometric parameters, TCD was seen to demonstrate the best correlation with GA. In their study of 192 women, Nikolov et al. discovered a significant association between GA and TCD (r = 0.980), BPD (r = 0.960), and FL (r = 0.980). They suggest that TCD should be used as a standard method for determining the GA of the fetuses [15]. These observations exhibit similarities to the present research. The research conducted by Meyer et al. showed a significant correlation between TCD and GA (r^2 ^= 0.9464), AC and GA (r^2 ^= 0.9685), and TCD and AC (r^2 ^= 0.9561) [16]. These observations bear a resemblance to the current research.

In their research of 50 antenatal women who were in the 14-40 weeks of gestation, Goel et al. discovered that TCD showed a significant association with GA (r = 0.991, p < 0.001). Compared to other USG parameters, TCD showed a significant association with menstrual age (r = 0.9840, p = 0.0001) [17]. In a research carried out by Dashottar et al., it was found that the accuracy of estimating GA using TCD was significantly higher during the third trimester (after 28 weeks) compared to the second trimester (between 16 and 27 weeks). However, the overall estimates of GA strongly correlated with the actual GA (r = 0.9840), indicating no statistically significant difference between the estimated and actual GA. Thus, this study further supports the finding that TCD in the third trimester of pregnancy was a stronger predictor of GA in pregnancies with both normal and IUGR fetuses [18]. These observations bear a resemblance to the current research.

The current research establishes a linear relationship between TCD and advancing GA in fetuses with normal growth. Research conducted by Chavez et al. demonstrated identical results. This could be due to the fact that TCD is minimally affected by fetal growth restriction, supporting the hypothesis that the brain-sparing effect facilitates human cerebellar growth and makes it resistant to chronic hypoxemia [13].

The current research demonstrates a strong association between TCD and BPD (r = 0.9731; p = 0.0001), between TCD and HC (r = 0.9643; p = 0.0001), between TCD and AC (r = 0.9617; p = 0.0001), and between TCD and FL (r = 0.9868; p = 0.0001). Among these parameters, FL shows a good correlation (r = 0.9868; p = 0.0001) with TCD (Table 6). In their study of 130 antenatal women, George et al. discovered a significant association between TCD and BPD (r^2 ^= 0.992), HC (r^2 ^= 0.991), AC (r^2 ^= 0.993), and FL (r^2 ^= 0.996). Of these parameters, FL shows a strong association with TCD [19]. These observations are identical to the current research.

The current research establishes a significant linear relationship between TCD and GA, with a correlation coefficient of 0.9865 and a p-value of 0.0001. The research conducted by Chavez et al., Goel et al., George et al., Goldstein et al., and Kumar et al. demonstrated comparable findings [13,17,19-21] (Table 7).

**Table 7:** Comparison of correlation coefficient of the current study with previous studies GA: gestational age; TCD: transcerebellar diameter

Study	Correlation coefficient between GA and TCD	p-value
Chavez et al. [13]	0.9500	0.001
Goel et al. [17]	0.9910	<0.001
George et al. [19]	0.9950	<0.001
Goldstein et al. [20]	0.9480	0.0001
Kumar et al. [21]	0.9930	<0.001
Current study	0.9865	0.0001

In the current research, TCD showed a correct assessment of GA by the LMP in 479 (95.8%) out of 500 antenatal women. In an investigation of 100 pregnant women in the GA range of 16-40 weeks, Malik et al. discovered that TCD had a prediction accuracy of 92%, which was higher than the standard nomogram developed by Malik et al. [22]. The research carried out by Chavez et al. and Naseem et al. demonstrated comparable results [13,23] (Table 8).

**Table 8:** Comparison of the accuracy of TCD in the current study with previous studies TCD: transcerebellar diameter; GA: gestational age

Study	Accuracy of TCD in predicting GA
Chavez et al. [13]	94.0%
Malik et al. [22]	92.0%
Naseem et al. [23]	91.7%
Current study	95.8%

The findings of the present research, along with the findings of the other studies that have been stated, indicate that TCD can be utilized as an independent measure to determine GA in the second and third trimesters of pregnancy, particularly in cases where the last menstrual period is unknown, no dating scan has been performed, or in fetuses with variations in head shape such as dolichocephaly and brachycephaly.

During the late third trimester of pregnancy, it was observed that some pregnant women experienced discomfort when rocking the probe on the fetal head to obtain the sub-occipito-bregmatic view for the TCD measurement. Additionally, shadows from the fetal skull prevented some antenatal women from having a clear view of the edges of the cerebellum. There were a few patients in whom the fetus was highly active, and we experienced some difficulties while we were attempting to measure the TCD using an USG machine. We acknowledge that the TCD measurements may have been unsatisfactory in the aforementioned circumstances, even though the percentage was less than 2%.

It is important to note that the current research does have certain inherent limitations. The research was conducted on a small group of pregnant women recruited from a single institution. This investigation was conducted for a relatively brief period of time. Antenatal women with anomalous babies, multiple gestations, unreliable dates, and those suspected to have IUGR were not included in the current study. On the other hand, the adoption of research projects that are more comprehensive and involve a larger cohort has the potential to produce a more accurate portrayal of the issue that is being investigated.

## Conclusions

The current research demonstrated a significant linear relationship between TCD and GA, as well as a strong association between GA and BPD, HC, AC, and FL in the second and third trimesters of pregnancy. The TCD showed the best correlation with GA among all the biometric parameters. The current research concludes that the TCD can be utilized as an independent measure to determine GA in the second and third trimesters of pregnancy, particularly in cases where the LMP is unknown, no dating scan has been performed in the first trimester, the initial assessment takes place in the third trimester, and in fetuses with variations in head shape such as dolichocephaly and brachycephaly.

## References

[1] Cunningham FG, Leveno KJ, Bloom SL (2010). Williams Obstetrics.

[2] Mandruzzato G, Antsaklis A, Botet F (2008). Intrauterine restriction (IUGR). J Perinat Med.

[3] Agrawal C, Agrawal KK, Gandhi S (2016). Assessment of fetal growth using the ratio of the transverse cerebellar diameter to abdominal circumference. Int J Gynaecol Obstet.

[4] Srividya R, Himavarshini K, Pathri M (2022). Evaluation of transverse cerebellar diameter/abdominal circumference ratio-in assessing fetal growth restriction. Int J Acad Med Pharm.

[5] Leitner Y, Fattal-Valevski A, Geva R (2007). Neurodevelopmental outcome of children with intrauterine growth retardation: a longitudinal, 10-year prospective study. J Child Neurol.

[6] Nardozza LM, Caetano AC, Zamarian AC (2017). Fetal growth restriction: current knowledge. Arch Gynecol Obstet.

[7] Hashimoto K, Shimizu T, Shimoya K, Kanzaki T, Clapp JF, Murata Y (2001). Fetal cerebellum: US appearance with advancing gestational age. Radiology.

[8] Meyer WJ, Gauthier DW, Goldenberg B, Santolaya J, Sipos J, Cattledge F (1993). The fetal transverse cerebellar diameter/abdominal circumference ratio: a gestational age-independent method of assessing fetal size. J Ultrasound Med.

[9] Manning FA (1990). The use of sonography in the evaluation of the high-risk pregnancy. Radiol Clin North Am.

[10] Bhimarao Bhimarao, Nagaraju RM, Bhat V, Gowda PV (2015). Efficacy of Transcerebellar diameter/abdominal circumference versus head circumference/abdominal circumference in predicting asymmetric intrauterine growth retardation. J Clin Diagn Res.

[11] Maryi SS, Indirani D, Vinothkumar PS (2022). Efficacy of trans-cerebellar diameter/abdominal circumference ratio versus head circumference/ abdominal circumference ratio in prediction of asymmetrical intrauterine growth retardation. Int J Reprod Contracept Obstet Gynecol.

[12] Vinkesteijn AS, Mulder PG, Wladimiroff JW (2000). Fetal transverse cerebellar diameter measurements in normal and reduced fetal growth. Ultrasound Obstet Gynecol.

[13] Chavez MR, Ananth CV, Smulian JC, Yeo L, Oyelese Y, Vintzileos AM (2004). Fetal transcerebellar diameter measurement with particular emphasis in the third trimester: a reliable predictor of gestational age. Am J Obstet Gynecol.

[14] Liu F, Zhang Z, Lin X (2011). Development of the human fetal cerebellum in the second trimester: a post mortem magnetic resonance imaging evaluation. J Anat.

[15] Nikolov V, Khadzhiev A, Brankova M, Novachkov V (1991). The echographic measurement of fetal transverse cerebellar diameter in the second pregnancy trimester - a "nonstandard" method for determining gestational age (Article in Bulgarian). Akush Ginekol (Sofiia).

[16] Meyer WJ, Gauthier D, Ramakrishnan V (1994). Ultrasonographic detection of abnormal fetal growth with the gestational age-independent, transverse cerebellar diameter/abdominal circumference ratio. Am J Obstet Gynecol.

[17] Goel P, Singla M, Ghal R (2010). Transverse cerebellar diameter - a marker for estimation of gestational age. J Anat Soc India.

[18] Dashottar S, Senger KPS, Shukla Y (2018). Transcerebellar diameter: an effective tool in predicting gestational age in normal and IUGR pregnancy. Int J Reprod Contracept Obstet Gynecol.

[19] George R, Amirthalingam U, Hussain MRK (2021). Can trans-cerebellar diameter supersede other fetal biometry in measuring gestational age? A prospective study. Egypt J Radiol.

[20] Goldstein I, Reece EA, Pilu G (1987). Cerebellar measurements with ultrasonography in the evaluation of fetal growth and development. Am J Obstet Gynecol.

[21] Kumar M, Kaushik R, Gupta D (2020). Transverse cerebellar diameter as an independent predictor of gestational age in normal and IUGR pregnancies. Int J Contemp Med Surg Radiol.

[22] Malik R, Pandya VK, Shrivastav P (2003). Gestational age estimation using trans-cerebellar diameter with grading of fetal cerebellum and evaluation of TCD/AC ratio as a gestational age independent parameter. Indian J Radiol Imaging.

[23] Naseem F, Fatima N, Yasmeen S, Saleem S (2013). Comparison between transcerebellar diameter with biparietal diameter of ultrasound for gestational age measurement in third trimester of pregnancy. J Coll Physicians Surg Pak.

